# Clinical Presentations and Risk Factors of Gastrointestinal Bleeding in the Emergency Department: A Multicenter Retrospective Study

**DOI:** 10.7759/cureus.59912

**Published:** 2024-05-08

**Authors:** Abed H AlLehibi, Faisal F Alsubaie, Rayan H Alzahrani, Hussain A Ekhuraidah, Mohammed A Koshan, Nasser F Alotaibi, Fahad M Alotaibi, Hamdan S Alghamdi, Abdulrahman A Aljumah

**Affiliations:** 1 Gastroenterology, King Fahad Medical City, Ministry of Health, Riyadh, SAU; 2 Clinical Sciences, College of Medicine, Dar Al Uloom University, Riyadh, SAU; 3 College of Medicine, King Saud Bin Abdulaziz University for Health Sciences, Ministry of National Guard Health Affairs, Riyadh, SAU; 4 King Abdullah International Medical Research Centre, Ministry of National Guard Health Affairs, Riyadh, SAU; 5 Hepatology Section, Hepatobiliary Sciences and Organ Transplant Centre, King Abdulaziz Medical City, Ministry of National Guard Health Affairs, Riyadh, SAU

**Keywords:** emergency department, polyps, colonoscopy, gastritis, varices, endoscopy, hematemesis, melena, gastrointestinal bleeding

## Abstract

Background and aims: Gastrointestinal bleeding is a major healthcare burden and is associated with significant morbidity and mortality. This study aimed to assess the prevalence, clinical presentation, and risk factors of patients presenting with gastrointestinal bleeding in the emergency department.

Materials and methods: This retrospective study was conducted in two tertiary care hospitals in Riyadh, Saudi Arabia. The medical records of patients who presented to the emergency department with gastrointestinal bleeding between January 2010 and January 2020 were reviewed. Patients aged 18 years or older, with gastrointestinal bleeding (upper or lower) regardless of underlying cause, lifestyle, location of bleeding, health status, or medication use, were included. Demographic characteristics, initial vital signs, medical history, physical examination findings, comorbidities, medications, laboratory and radiological investigations, cause and stage of liver disease, management, and complications were recorded. Endoscopic findings and management of the bleeding site were collected according to the presenting symptoms.

Results: A total of 760 patients were included. The mean age was 62.7 ± 17.8 years, and 61.4% were males. The most common comorbidities at presentation were hypertension (54.1%), diabetes mellitus (51.2%), and ischemic heart disease (18.2%). The origins of the bleeding were lower gastrointestinal in 52% and upper gastrointestinal in 48% of patients.

Conclusions: Lower gastrointestinal bleeding was found to be more common than upper gastrointestinal bleeding. Hemorrhoids, polyps, diverticular disease, and colonic ulcers were the major risk factors for lower gastrointestinal bleeding. In contrast, upper gastrointestinal bleeding was predominantly caused by esophageal varices, gastritis, and peptic ulcers.

## Introduction

Despite recent advancements in healthcare, gastrointestinal bleeding (GIB) remains a life-threatening condition in both emergency and inpatient settings [1-3]. GIBs are categorized into two types: upper and lower, related to anatomical landmarks such as the ligament of Treitz. In the United States, the incidence of upper GIB is estimated at 50-100 per 100,000 persons per year, with a lower GIB incidence of approximately 21 per 100,000 persons per year [4]. About 80% to 85% of GIB cases resolve spontaneously without any intervention or with almost negligible interference; however, these episodes may cause major and extensive bleeding, leading to eventual death [5,6].

The clinical presentation of upper GIB is vomiting of blood: fresh red (hematemesis), altered dark red (coffee-ground emesis), and dark, tar-colored stools (melena). Hematochezia is the passage of bright red blood per anus, usually with stools. It can occur from bleeding anywhere in the lower gastrointestinal (GI) tract and not only from the rectum and may also occur with massive upper GIB [3]. Lower GIB classically presents with hematochezia; however, bleeding from the right colon or small intestine may cause melena. Left colon bleeding tends to be frank red [3]. Additionally, patients with small amounts of bleeding, particularly the elderly, may present with complaints such as weakness, fatigue, or syncope.

In a study published in 2021, the etiological causes for upper GIB were found to be non-variceal in 80.1% (95% CI: 75.4%-85.3%) and variceal in 19.91% (95% CI: 15.25%-25.16%) of cases. Active bleeding was found in 6.58% [7]. Studies from Saudi Arabia reported a 38% to 45% variceal cause of upper GIB [8-11]. Over the years, the prevalence of *Helicobacter pylori* has changed. Older reports showed that the prevalence of *H. pylori* was about 40%-50% in the children age group and 70% in the age group older than 20 years [12]. Recent reports revealed a prevalence range from 28% to 46% among adults in different geographical regions of Saudi Arabia [13-16]. Additionally, the prevalence of *H. pylori* in Saudi children has dropped significantly to 18.2% [17]. Older age seems to have more prevalence in certain geographical areas of Saudi Arabia reaching up to 67% which increases with age [18]. The decline may be related to changes in socioeconomic, lifestyle, environmental factors, and the method of detection [16]. It is also higher in symptomatic individuals compared to healthy subjects [14,16]. Although the recent studies reported declined prevalence compared to older ones, it must be noted that the older studies were primarily using single serological tests. With the improvement of the serological tests and the introduction of urea breath tests and histology, it is expected that the detection rate will change. This change also has been attributed to changes in endoscopic lesions identified on esophagogastroduodenoscopy (EGD) in patients with dyspepsia over time [19,20]. The variability in the proportion of causes of upper GIB across geographical regions may be influenced by the prevalence of *H. pylori* and viral hepatitis [17,21]. The demographic features of populations that reflect the burden of non-communicable diseases and their associated morbidities may have also contributed to the several causes of GIB [22,23].

Diverticular disease accounts for 50% of lower GIB cases, followed by colitis and anorectal lesions [24]. The major predisposing risk factors have been reported to be related to age, comorbidities, history of GIB, and the use of anticoagulants and antiplatelet drugs [25]. While there have been several studies conducted on GIB in the region, our study is unique in its focus on identifying the prevalence, clinical presentation, and risk factors for GIB in patients specifically presenting to emergency departments [1,26]. Additionally, while previous studies have reported on the prevalence and risk factors of GIB in Saudi Arabia, there is limited research on the clinical presentation of GIB in this population. Therefore, our study aims to assess the clinical presentation, risk factors, and prevalence of patients presenting with GIB in the emergency department of tertiary care centers in Saudi Arabia.

## Materials and methods

This retrospective multicenter study was conducted in two large tertiary hospitals (King Fahad Medical City and King Abdulaziz Medical City) in Riyadh, Saudi Arabia, and included patients older than 18 years of age who visited the emergency department and were admitted to hospital with GIB and underwent upper or lower GI endoscopy between January 1, 2010 and January 1, 2020.

We included all patients who presented to the emergency department with any form of upper or lower GI bleeding (such as melena, hematochezia, or hematemesis) and then underwent endoscopy to confirm the diagnosis and identify the source of bleeding. Patients with no available endoscopy records or who did not require hospital admission and those who underwent endoscopic procedures for another reason than GIB were excluded from the study. Patients younger than 18 years of age were also excluded.

Patient demographic details, vital signs, medical and surgical history, clinical presentation, comorbidities, medication history (proton pump inhibitors (PPI), non-steroidal anti-inflammatory drugs (NSAIDs), antiplatelets, and anticoagulants), laboratory results (blood glucose levels, complete blood count, blood chemistry, coagulation profile, inflammatory markers, and liver function tests), liver disease etiology and stage, management (intensive care unit (ICU) admission and length of stay, the in-hospital mortality rate, blood product transfusion, history of rebleeding within one week of the first bleeding event, patients treated and discharged within one week, and endoscopy findings (EGD, colonoscopy, and endoscopy capsule) were collected from their medical records.

Ethical considerations

This study was approved by the institutional review boards of the included hospitals, which waived the need for consent because of the retrospective nature of this study. The study was conducted in accordance with the 2010 updated Declarations of Helsinki.

Statistical analysis

The data analyzed various variables, including both categorical and continuous types. Categorical variables, such as gender, nationality, marital status, smoking, drinking, ethnicity, etc., were presented as frequencies and percentages. Meanwhile, continuous variables, such as age, weight, height, BMI, etc., were presented as mean ± SD, while non-normally distributed variables, such as blood glucose, red blood cells, etc., were expressed as median (interquartile range (IQR)). The normal distribution assumption was confirmed via the Kolmogorov-Smirnov test, and non-parametric tests were utilized in the case of biased data. Pearson chi-square/Fisher's exact test was deployed to determine significant associations between categorical variables, with a two-sided p-value of less than 0.05 considered statistically significant. The Mann-Whitney U test/independent sample t-test was conducted to compare both bleeding groups with laboratory investigations and the patient's clinical characteristics. The data was analyzed using the SPSS 25 Statistics Package (SPSS Inc., Chicago, Illinois, USA) MEDCALC version 18.11.6 (Acacialaan 22 8400 Ostend, Belgium).

## Results

A total of 760 patients (642 from King Abdulaziz Medical City, 118 from King Fahad Medical City) who underwent an EGD for evaluation of acute GIB were included in the study. Patient demographic data included a mean age of 62.69 ± 17.81 years (range: 18-109 years); 467 (61.4%) males and 293 (38.6%) females; a mean body mass index of 27.983 ± 7.20; and 99.2% Arabian ethnicity. Saudi nationals comprised 97.2% of the study population (97% from Riyadh and 3% from other regions). Among the patients, 3.7% were smokers, 1.3% had a history of alcohol consumption, 86.1% were married, 2.4% had a history of portal vein thrombosis, 6.2% had a history of peptic ulcer disease, and 13.7% had known esophageal varices. Table 1 demonstrates the demographic and clinical characteristics of the study population.

**Table 1:** Demographic and Clinical Characteristics of Patients The categorical data are presented as frequency (%), and the continuous data are presented as mean ± standard deviation. GI: gastrointestinal.

Variables		Mean ± SD	Frequency (%)
Age (years)		62.69 ± 17.81	
Weight (kg)		72.83 ± 18.87	
Height (cm)		161.59 ± 10.58	
Pulse rate		85.98 ± 17.91	
Systolic blood pressure		124.15 ± 23.79	
Diastolic blood pressure		67.15 ± 14.96	
Body temperature		36.8 ± 2	
Respiratory rate		20.37 ± 3.15	
Body mass index		27.98 ± 7.20	
Gender	Male		467 (61.4%)
	Female		293 (38.6%)
Marital status	Married		654 (86.1%)
	Single		68 (8.9%)
	Unknown		38 (5.0%)
Social habits	Smoking		28 (3.7%)
	Alcohol intake		10 (1.3%)
Associated risk factors	Peptic ulcer		47 (6.2%)
	Esophageal varices		104 (13.7%)
	Portal vein thrombosis		18 (2.4%)
Presentation of GI bleeding	Melena		320 (42.1%)
	Hematemesis		164 (21.6%)
	Hematochezia		244 (32.1%)
	Combined presentation		144 (18.9%)
Clinical features	Abdominal pain		289 (38.0%)
	Vomiting		148 (19.5%)
	Dizziness		97 (12.8%)
	Hepatomegaly		22 (2.9%)
	Splenomegaly		33 (4.3%)
	Peripheral edema		76 (10.0%)
	Ascites		133 (17.5%)

The initial laboratory tests revealed a low mean hemoglobin level at 103.25 ± 35.34 and low iron with a median of 6.5, IQR (11-3.37). Median platelets and white blood cells were within normal range. The means of the liver and renal profile were within normal. The results of the laboratory investigations obtained at the time of the patient's presentation to the emergency department are displayed in Table 2.

**Table 2:** Initial Laboratory Investigations Continuous data are presented as mean ± SD and median, interquartile range (IQR).

Parameters	Normal Values	Mean ± SD	Median (IQR)
Hematology			
Red blood cells	4.5-6.1 x 10^12^/L		3.75 (4.518-2.96)
Hemoglobin	135-180 g/L	103.25 ± 35.34	
Hematocrit	0.42-0.54		0.32 (0.391-0.267)
Mean corpuscular volume	76-96 fL	87.73 ± 10.99	
Erythrocyte sedimentation rate	0-20 mm/h		39 (71-16)
Platelet count	150-400 x 10^9^/L		236 (321-145)
White blood cells	4-11 x 10^9^/L		7.6 (10.4-5.7)
Coagulation profile			
Prothrombin time	9.38-12.34 s		12.2 (14.5-11.25)
Partial thromboplastin time	24.84-32.96 s		29.2 (34-26.5)
International normalized ratio	0.80-1.20		1.12 (1.32-1.02)
Biochemistry			
Blood glucose level	4.6-6.4 mmol/L		6.9 (9.8-5.5)
Sodium level	136-145 mmol/L		137 (139-134)
Potassium level	3.5-5.1 mmol/L	4.33 ± 0.76	
Creatinine	64-110 µmol/L		76 (121-61)
Blood urea nitrogen	3-9.2 mmol/L		6.5 (12-3.9)
C-reactive protein	0-8 mg/L		21 (76.5-5)
Iron	11-29 µmol/L		6.5 (11-3.37)
Ferritin	4.6-204 ng/mL		86.7 (279.8-25)
Total iron-binding capacity	45-82 µmol/L	42.6 (57-31.5)	
Albumin	32-46 g/L	33.37 ± 7.67	
Total protein	64-83 g/L	63.29 ± 11.28	
Total bilirubin	5.1-20.5 µmol/L		11.3 (27.83-6.98)
Alkaline phosphatase	40-150 units/L		92.5 (148-67)
Alanine transaminase	5-55 units/L		19 (33-13)
Aspartate transaminase	5-34 units/L		24 (44-17)
Gamma-glutamyl transferase	9-50 units/L		83.45 (180.75-35)

Hypertension (54.1%), diabetes mellitus (51.2%), ischemic heart disease (18.2%), chronic liver disease (12.4%), malignancy (14.9%), cerebrovascular accident (10.1%), hepatitis C (5.5%), hepatitis B (4.6%), and chronic obstructive pulmonary disease (4.6%) constituted the most common comorbid conditions. Regarding medications, approximately half the study population (48.9%) had a history of proton pump inhibitor use, 29.6% had used aspirin, 25.8% heparin, 19.5% NSAIDS, 11.2% cyclooxygenase-2 (COX-2) inhibitors, 9.9% steroids, 8.7% warfarin, and 3.7% had used factor 10a inhibitors. In our cohort, 52% of subjects presented with lower GIB and 48% presented with upper GIB.

Symptoms at presentation included melena (42.1%), abdominal pain (38.0%), hematochezia (32.1%), hematemesis (21.6%), vomiting (19.5%), coffee-ground vomiting (12.8%), and dizziness (12.8%). On clinical examination, 17% of patients presented with ascites (7.4% grade 2, 4.3% grade 1, and 3.4% grade 3), 10% with peripheral edema, 4.3% with splenomegaly, and 2.9% with hepatomegaly. None of the patients presented with palmar erythema, caput medusa, or spider nevi on admission.

Vital signs on presentation were a mean pulse rate of 85.98 ± 17.91 beats/min, a systolic blood pressure of 124.15 ± 23.79 mmHg, a diastolic blood pressure of 67.14 ± 14.96 mmHg, a respiratory rate of 20.36 ± 3.14 breaths/min, and a body temperature of 36.80 ± 1.99°C. The mean blood transfusion rate for patients who received packed red blood cells (PRBCs) was 1.13 ± 2.34 units and 0.5 ± 2.62 units for fresh frozen plasma.

A total of 171 patients were admitted to ICU, with a mean duration of the ICU stay of 10.63 ± 12.23 days (range: 2-79 days). The frequencies of rebleeding events within one week from the first bleeding event were zero for 82.0%, six or more for 10.3%, two for 3.0%, three for 2.1%, one for 1.3%, four for 1.3%, and five for 0.1% of patients with overall rate of rebleeding of 18%. Additionally, in-hospital mortality was 12.1%, whereas 28.0% of patients were treated and discharged within a week.

Comparison between upper and lower GIB revealed that 69 (19.0%) cases of upper and 144 (36.5%) cases of lower GIB, p-value ≤ 0.001, were treated and discharged within one week of the event of bleeding. However, 65 (17.8%) cases of upper and 27 (6.8%) cases of lower GIB, p-value ≤ 0.001, had in-hospital mortality within one week of the event of bleeding. There was a tendency toward older age to have upper GIB compared to lower GIB; 66.54 ± 16.73 years for upper GIB versus 59.12 ± 18.06 years for lower GIB, p-value ≤ 0.001. The comparison of the association and impact of certain variables between patients with upper and lower gastrointestinal bleeding is displayed in Table 3.

**Table 3:** Comparison of the Association and Impact of Variables Between Patients With Upper and Lower Gastrointestinal Bleeding GIB: gastrointestinal bleeding, SD: standard deviation, IQR: interquartile range, BMI: body mass index, NSAIDs: non-steroidal anti-inflammatory drugs, COX-2: cyclooxygenase-2. The categorical data are presented as frequency (%), while continuous data are presented as mean ± SD and Median (IQR). *P-value is significant at p<0.05.

Variables	Description	Source of GIB		P-value
		Upper	Lower	
		(n = 365)	(n = 395)	
Demography				
Gender	Male	230 (63.0%)	237 (60.0%)	0.394
	Female	135 (37.0%)	158 (40.0%)	
Age (years)	Mean ± SD	66.54 ± 16.73	59.12 ± 18.06	*<0.001
Height	Mean ± SD	160.77 ± 10.87	162.33 ± 10.29	0.055
Weight	Mean ± SD	69.9 ± 18.1	75.46 ± 19.19	*<0.001
Body mass index (BMI)	Mean ± SD	27.1 ± 7.23	28.77 ± 7.1	*0.002
BMI classifications	Normal (< 24.9)	142 (38.9%)	110 (27.8%)	*0.001
	Abnormal (≥ 25)	223 (61.1%)	285 (72.2%)	
Social habits				
Smoking	Frequency (%)	13 (3.6%)	15 (3.8%)	0.863
Alcohol intake	Frequency (%)	8 (2.2%)	2 (0.5%)	*0.042
Associated medications				
Aspirin	Frequency (%)	114 (31.2%)	111 (28.1%)	0.345
Steroid	Frequency (%)	37 (10.1%)	38 (9.6%)	0.811
NSAIDs	Frequency (%)	93 (25.5%)	55 (13.9%)	*0.001
COX-2 inhibitor	Frequency (%)	37 (10.1%)	48 (12.2%)	0.379
Warfarin	Frequency (%)	40 (11.0%)	26 (6.6%)	*0.032
Heparin	Frequency (%)	110 (30.1%)	86 (21.8%)	*0.008
Factor Xa inhibitors	Frequency (%)	14 (3.8%)	14 (3.5%)	0.831
Proton pump inhibitors	Frequency (%)	196 (53.7%)	176 (44.6%)	*0.012
Associated comorbidity				
Hypertension	Frequency (%)	213 (58.4%)	198 (50.1%)	*0.023
Diabetes	Frequency (%)	207 (56.7%)	182 (46.1%)	*0.003
Ischemic heart disease	Frequency (%)	77 (21.1%)	61 (15.4%)	*0.043
Chronic liver disease	Frequency (%)	69 (18.9%)	25 (6.3%)	*<0.001
Chronic pulmonary disease	Frequency (%)	22 (6.0%)	13 (3.3%)	0.072
Laboratory profile				
Hemoglobin	Mean ± SD	93.89 ± 36.65	111.87 ± 31.78	*<0.001
Blood glucose level	Median (IQR)	7.65 (10.7-6)	6.3 (8.68-5.2)	*<0.001
Hematocrit	Median (IQR)	0.3 (0.35-0.24)	0.36 (0.42-0.3)	*<0.001
Platelet count	Median (IQR)	182 (295.5-114)	259 (334-205)	*<0.001
White blood cells	Median (IQR)	8.17 (11.28-5.9)	7.3 (9.63-5.6)	*0.009
Prothrombin time	Median (IQR)	13 (16.4-11.6)	11.6 (12.73-10.9)	*<0.001
Partial thromboplastin time	Median (IQR)	30.7 (37.1-27)	28.4 (31.3-26.2)	*<0.001
International normalized ratio	Median (IQR)	1.21 (1.49-1.08)	1.07 (1.17-1)	0.163
Outcome				
Treated and discharged	Frequency (%)	69 (19.0%)	144 (36.5%)	*<0.001
In-hospital mortality	Frequency (%)	65 (17.8%)	27 (6.8%)	*<0.001

Chronic liver disease was found in 123 patients (16.2% of the total subjects). The causes were as follows: hepatitis B, 34 (4.5%); hepatitis C, 38 (5.0%); alcohol, four (0.5%); autoimmune, nine (1.2%); Wilson’s disease, one (0.1%); schistosomiasis, five (0.7%), and non-alcoholic fatty liver disease, 32 (4.2%). The majority of chronic liver disease patients were Child-Pugh class B (6.6% of the study population), 5.4% were Child-Pugh class C, and 4.5% were Child-Pugh class A.

The most common findings on endoscopy for upper GIB were esophageal varices (18.8%). The frequencies of other findings were 14.5% gastritis, 12.8% peptic ulcer disease, 3.4% esophagitis, 2.9% tumors, 2.0% gastric varices, 0.5% portal hypertensive gastropathy, 0.4% esophageal (Mallory-Weiss) tears, 0.1% arteriovenous malformations, and 10.3% other causes. Endoscopic results were negative for any pathologic findings in 27.5% of the study population.

The most common findings on colonoscopy for those with lower GIB were hemorrhoids (21.4%). The frequencies of other findings were 11.1% polyps, 8.8% diverticula, 7.6% ulcers, 3.6% tumors, 2.5% carcinomas, 1.7% angiodysplasias, 0.7% varices, and 4.9% other causes. Endoscopy was negative for 21.1% of the study population.

## Discussion

In this current study, we aimed to evaluate the clinical presentation and risk factors of patients presenting with GIB in the emergency department of tertiary care centers in Saudi Arabia. The most common findings in our study were compared with those of similar local and international studies. Melena was the most common presentation in our population (42.1%), while in the study by Almadi et al., it accounted for 66.2% of the other common symptoms, such as abdominal pain and hematemesis [7]. In the study by Almadi et al., the in-hospital mortality rate was 4.4% compared with the 12.1% in our study. Both our study and that of Almadi et al. showed that hypertension was one of the frequent prevalent comorbidities among patients with GIB [7]. El-Tawil stated that esophageal varices accounted for 10% of upper GIB with mortality rates ranging from 30% in the first episode to 60% in subsequent episodes [27]. Furthermore, Kim et al. highlighted that lower GIB is significantly associated with advanced age and more than two comorbidities, with a mortality rate ranging between 2.4% and 3.9% [3]. In our study, lower GIB was more in younger ages and had less mortality than upper GIB. There were no significant differences in terms of associated comorbidities between upper and lower GIB except for chronic liver disease which is more common in upper GIB.

Among patients with lower GIB, the most common symptom reported by Alruzug et al. was hematochezia, with a prevalence of approximately 90%, compared with our study with only 32.1% [28]. This is possible because some hematochezia cases are due to minor rectal bleeding caused by hemorrhoids that do not require endoscopy and can be managed as an outpatient; hence, we have excluded them from our study. As per our inclusion criteria, all our subjects must have a mode of endoscopy as an inpatient to be included.

In our study, the second most prevalent associated finding for GIB was the presence of esophageal varices, found in 13.7% of our patients. According to a recent study, 18.9% of patients who were presented with GIB had a history of esophageal varices diagnosed during a prior endoscopy [29]. We would like to address the importance of this pathology as a prevalent predisposing cause for GIB and encourage varices screening in patients with chronic liver disease and watch for its development by primary and secondary prevention of variceal bleeding.

The mean duration of ICU stay was 10.63 ±12.23 days which is much shorter than what has been reported by another local study by Almadi et al., in which the mean duration of stay was 25 ± 34.16 days. However, in our study, the percentage of patients who received blood transfusions from the total cases of GIB was higher at 32.1%, in contrast to Almadi et al. where 13.9% of their population received PRBCs. The mean rate of blood transfusion was similar in our study compared to Almadi et al., at 3.5 ± 2.9 units and 3.53 ± 0.97 units, respectively [7].

Moreover, the frequencies of rebleeding events were zero for 82.0%, six or more for 10.3%, two for 3.0%, three for 2.1%, one for 1.3%, four for 1.3%, and five for 0.1% of patients. However, the rebleeding rate was 18% in comparison to 8.9% in the study by Almadi et al., which revealed that the rebleeding frequency among our patients was higher than in similar studies [7].

The reason why we had a higher rebleeding rate compared to Almadi et al. is not quite known. However, this probably is because our patient population had a relatively older mean age (62.7 vs. 57.1 years). Moreover, the lesions identified on endoscopy in our cohort had a lower percentage of peptic ulcer disease (12.8% vs. 36%), esophagitis/gastroesophageal reflux disease (GERD) (3.4% vs. 38.1%), and gastric varices (2% vs. 22%) compared with those described by Almadi et al. [7]. It is also possible that this was related to the total number of patients. In Almadi et al., they have a total of 259 patients, while our cohort was 760 patients. Further, in Almadi et al., all their patients were of upper GIB only [7]. We have a significant number of lower GIB cases, and this has been reported to be associated with a higher recurrence rate when compared with upper GIB [2]. Finally, it could be that the rebleeding is due to an obscure GIB that was not identified during upper and lower GI endoscopic evaluation. The obscure bleeding is typically from the small intestine [3]. However, our result is comparable to other studies that have reported a rebleeding rate of 22.6% [30]. Furthermore, we found the prevalence of lower GIB during the 10-year study period to be 52%, in comparison with upper GIB accounting for 48%. According to a study published by Lanas et al., there was a trend toward increasing in the rate of lower GIB and a decrease in the upper GIB [2].

The findings of this study have important implications for clinical practice and future research. The higher prevalence of lower GIB compared to upper GIB underscores the need for increased attention to lower gastrointestinal tract pathology in the clinical evaluation and management of GIB. The identification of specific predisposing factors for lower and upper GIB can aid clinicians in making prompt and accurate diagnoses and implementing appropriate treatment strategies. Additionally, the high in-hospital mortality among these patients highlights the need for effective interventions to improve outcomes in this population. Future research should focus on developing and evaluating targeted interventions for specific underlying causes of GIB, as well as on identifying factors that can predict and prevent adverse outcomes.

Our study has the strength of being unique in its focus on identifying the prevalence, clinical presentation, and risk factors for GIB in patients specifically presenting to emergency departments. Additionally, our study has the strength of having comprehensive clinical and endoscopic data of patients presenting with upper or lower GIB allowing for optimal assessment of all etiological possibilities for GIB and not limited to certain pathology. The other important advantage is that our study is multicenter; hence, we avoided the possibility of selection bias; furthermore, it has a reasonably large number of patients. Finally, it is worth mentioning that our study has a low incidence of alcohol-related liver disease and a lower incidence of Mallory-Weiss syndrome.

However, like any study, there are some limitations that include the lack of specified timing for performing certain laboratory tests, endoscopic procedures, and proper documentation of data between the two centers due to the nature of the retrospective design. This has led to the exclusion of a substantial number of patients from enrollment in this study. Examples of exclusions are unclear endoscopy reports, incomplete demographic data, lack of documenting presence or absence of comorbidities, missing laboratory results, missing the name of medications including anticoagulants used by the patient, and proper timing of rebleeding. Another drawback is the lack of follow-up of patients after discharge. Our study, although elaborated on a unique finding namely low smoking and alcohol consumption among our patients, this may be considered as a drawback from advocating the generalizability of our data. Despite these limitations, our study highlighted the importance of GIB in this part of the world and the need for prospective studies.

## Conclusions

In our cohort, we found that lower GIB was more common than upper GIB. Hemorrhoids, polyps, diverticular disease, and colonic ulcers were the major associated factors for lower GIB. Upper GIB was predominantly caused by esophageal varices, gastritis, and peptic ulcers. Moreover, the most common symptoms included melena and abdominal pain. In-hospital mortality, rebleeding rate, and blood transfusion requirements were relatively high among our patients. On the other hand, the length of ICU stay was comparable to other studies.
